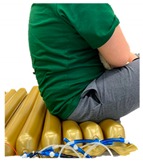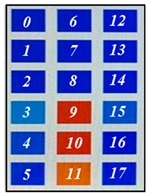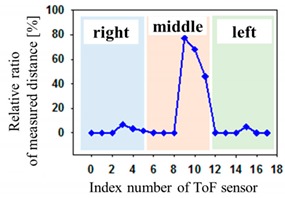# Active Body Pressure Relief System with Time-of-Flight Optical Pressure Sensors for Pressure Ulcer Prevention

**DOI:** 10.3390/s19183862

**Published:** 2019-09-06

**Authors:** Kang-Ho Lee, Yeong-Eun Kwon, Hyukjin Lee, Yongkoo Lee, Joonho Seo, Ohwon Kwon, Shin-Won Kang, Dongkyu Lee

**Affiliations:** 1 Daegu Research Center for Medical Devices, Korea institute of Machinery and Materials, Daegu 42994, Korea; 2 School of Electronics Engineering, College of IT Engineering, Kyungpook National University, Daegu 41566, Korea

**Keywords:** body pressure distribution, air-filled cell, alternating pressure air mattress, time of flight, air flow, active control system

## Abstract

A body pressure relief system was newly developed with optical pressure sensors for pressure ulcer prevention. Unlike a conventional alternating pressure air mattress (APAM), this system automatically regulates air flow into a body supporting mattress with adaptive inflation (or deflation) duration in response to the pressure level in order to reduce skin stress due to prolonged high pressures. The system continuously quantifies the body pressure distribution using time-of-flight (ToF) optical sensors. The proposed pressure sensor, a ToF optical sensor in the air-filled cell, measures changes in surface height of mattress when pressed under body weight, thereby indirectly indicating the interface pressure. Non-contact measurement of optical sensor usually improves the durability and repeatability of the system. The pressure sensor was successfully identified the 4 different-predefined postures, and quantitatively measured the body pressure distribution of them. Duty cycle of switches in solenoid valves was adjusted to 0–50% for pressure relief, which shows that the interface pressure was lower than 32 mmHg for pressure ulcer prevention.

## 1. Introduction

Generally, pressure ulcers are caused by a local breakdown of soft tissue due to prolonged high pressures at the interface of body and contact surface [1,2,3]. In order to relieve the interface pressure, manual repositioning of patients or support surfaces such as cushions has been required [1,2,3]. Recently, alternating pressure air mattresses (APAMs) have been often used for the prevention and treatment of pressure ulcers [4,5,6,7,8]. Air cells of APAMs sequentially inflate and deflate to relieve pressure for short periods. However, commercial available APAMs have a passive control mechanism that simply repeats inflation and deflation into air cells, which occurs regardless of the pressed regions and pressure level. It is difficult to show even and lower pressure distributions against different stress regions across the body.

To maximize the effect of the pressure relief through APAMs, a robust and reliable measurement of the body pressure is required simultaneously [1,5,8]. Usually, the main types of sensors to measure the interface pressure are based on the measurement of variations in resistance or capacitance from a deformable sensing component [9,10,11,12,13]. The capacitive pressure sensor measures changes in thickness between layers when pressed. Although the sensor has high sensitivity, it is subject to an interference environment due to the external electric field and requires complex readout circuitry such as a charge amplifier, a discharging resistor, and so on [12]. The resistive pressure sensor measures changes in conductivity of sensing material when pressed. This sensor has thin and flexible structures, and the sensor needs simple circuitry. However, its output signal is nonlinear with a slow response, and it shows high power consumption [13,14]. Both capacitive and resistive sensors have been reported that they have hysteresis limitation of the measured value drifts over time [14,15]. In addition, electric pressure sensors are usually weak in durability due to the deformation of a sensing element caused by physical contact force. The transducer placed on the contact surface across the body can also impose artificially high pressures on the tissues and poor resolution due to the thickness of the transducer [15,16]. As an alternative to the electric sensor, the optical sensor has provided good durability and external noise immunity [17,18,19]. Some device measured the pressure distribution for foot plantar using optic fiber [20]. In this study, a time-of-flight (ToF) optical sensor was used to measure the interface pressure. The ToF optical sensor identifies the distance away to the nearest object by measuring the time that the light takes to travel and reflect [21]. We implemented commercial ToF optical sensors on the bottom surface of air-filled cells in the mattress rather than the contact surface with body skin. The ToF optical sensor measures variations in the surface height of mattress after deformation. For the proof of concept, the optical pressure sensor visualized body pressure distribution and identified the 4 different postures. Here, we developed the APAM with an active control mechanism based on the ToF optical pressure sensor. This system has adaptive inflation (or deflation) duration of air mattress in response to the pressure level for body pressure relief of patient-specific interface regions. To investigate the performance of active body pressure relief system, we confirmed the rapid reduction of lower than 32 mmHg pressure using the system. It is expected that the proposed system can prevent pressure ulcers and improve sleep comfort and quality [22,23].

## 2. Materials and Methods

### 2.1. System Structure and Operation Principle

**Figure 1:**
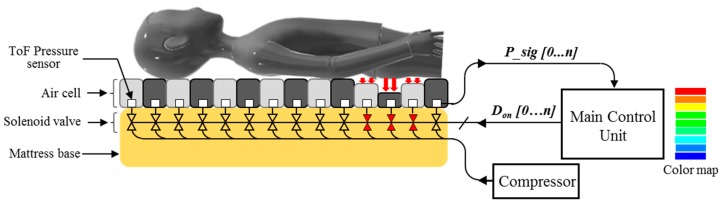
The schematic diagram of the proposed active pressure relief system with body pressure sensors.

Figure 1 shows a schematic diagram of the proposed active pressure relief system with body pressure sensors. The system includes a mattress with air-filled cells, ToF optical pressure sensors, solenoid valves, a main control unit, and a compressor. The top surface of a mattress has a lot of air-filled cells which directly support the human body. Here, the ToF optical pressure sensors are placed on the internal bottom surface of the air-filled cells, which means that these sensors are physically non-contacted from the interface surface of the body. The signals (*P_sig*) from the ToF optical sensors are collected together to the main control unit. The main control unit visualizes the body pressure distribution with the corresponding colors.

**Figure 2:**
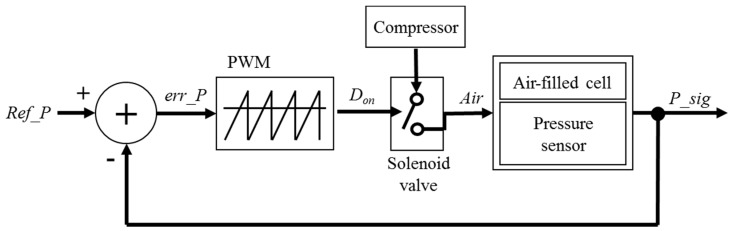
The block diagram of the closed-loop control mechanism in the system.

In the system, air flow is regulated by a negative feedback control mechanism. In other words, repetitive inflation and deflation duration in air-filled cell are determined by adjusting a duty cycle of solenoid valve switches in response to the magnitude of the measured pressure. As shown in Figure 1, if air-filled cells are strongly pressed (red arrows at the hip area), the corresponding solenoid valves with red color begin to regulate their air flow. Our system updates the pressure data after every cycle when air flow is at a steady-state. Therefore, we consider a simple, single-loop feedback system as shown in Figure 2. The output of the system *P_sig* is fed back with the unity-feedback gain and then, compared with the reference value *Ref_P*. The system periodically calculates error values as the difference between desired and measured pressure levels. The error value is analogue, classified by the predefined thresholds, and then duty cycle *D_on_* is changed. The *D_on_* describes the proportion of ON time to the regular interval. The *D_on_* is a result of pulse-width modulation (PWM) [24]. The output of the PWM is digital, switches the opening of the solenoid valves while regulating air flow applied by the compressor. In the system, the control loop uses a distance value as a feed-back variable. Therefore, the *Ref_P* means an initial distance without deformation and the measured distance is periodically compared with the *Ref_P* to determine the *D_on_* of switches in solenoid valves.

### 2.2. Design of the ToF Optical Pressure Sensor 

**Figure 3:**
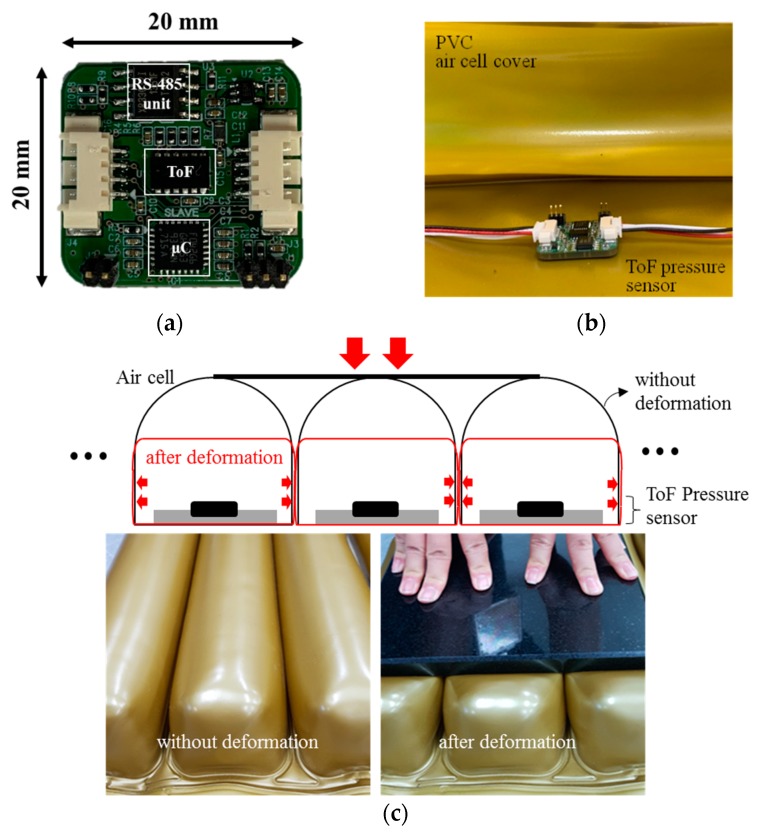
Pictures of (**a**) the developed ToF optical pressure sensor; (**b**) mounting the sensor inside of air-filled cell; (**c**) description of air-filled cell without and with deformation.

Figure 3a,b show pictures of the developed ToF optical pressure sensor and mounting inside of the air-filled cell, respectively. The ToF optical pressure sensor includes a microcontroller, a ToF unit and a communication component for external interface. As a ToF unit, we used an off-the-shelf component, VL6180X (STMicroelectronics, Plan-les-Ouates, Switzerland). In Figure 3c, air-filled cells on the mattress were closely arranged with a shape of semicircular cover on supporting walls. Therefore, when air-filled cells were pressed by the body, those were mainly deformed in vertical direction while leaning by each other. The ToF optical pressure sensor, which is placed on the bottom surface of air-filled cell, can measure the lowered height by using applied force. Therefore, we used the ToF optical sensor to indirectly measure the interface pressure under body weight. The ToF optical unit transfers its data to the microcontroller unit through the inter-integrated circuit bus (I2C bus). And both ends of the sensor share a common communication line that can be connected to other sensors. Although parallel sensors in other works may require a number of interface wires [9,11,25], our sensors are connected serially, minimizing the number of wires. The RS-485 communication technique needs only differential data lines for external interface [26]. All sensors can be independently separated with the assigned slave addresses. The sensor has a small size of 20(L) × 20(W) mm, which is suitable for using at any location as an independent module. It consumes a current of 30 mA from a 5 V supply.

**Figure 4:**
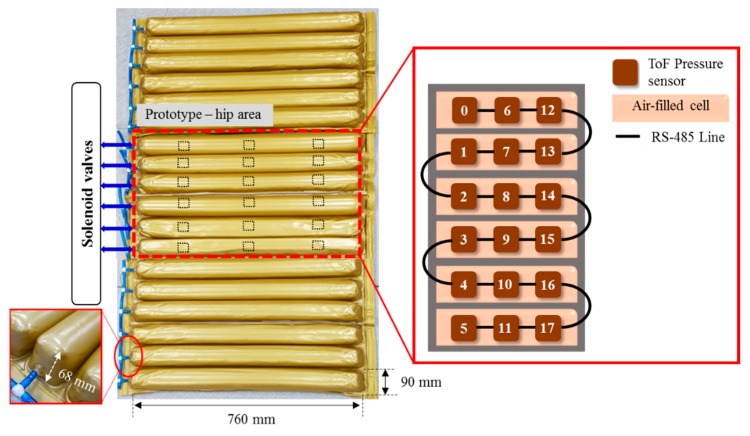
Description of pressure sensor location in air-filled cells of the mattress. The right inset shows the connection diagram of sensors with different addresses from 0 to 17. The left inset shows the hose pipe connected with the side of the air-filled cell.

Figure 4 shows the description of mounting pressure sensors in the air-filled cells of the mattress at a regular distance. The prototype was designed to fit the hip size of body, because the pressure is most focused on the hip of body parts [1,2]. The air-filled cell was fabricated with PVC (polyvinylchloride) film, which has a size of 760(L) × 90(W) × 68(H) mm. Total of 18 sensors in 6 air-filled cells of the prototype system were located. The 6 air-filled cells were arranged longitudinally. The serially connected 18 sensors have individual slave addresses of index numbers of 0–17 as shown in Figure 4. Hose pipe connects the side of the air-filled cells into the solenoid valve, as shown in the left inset of Figure 4.

### 2.3. Design of the Solenoid Valve System and Its Operation

**Figure 5:**
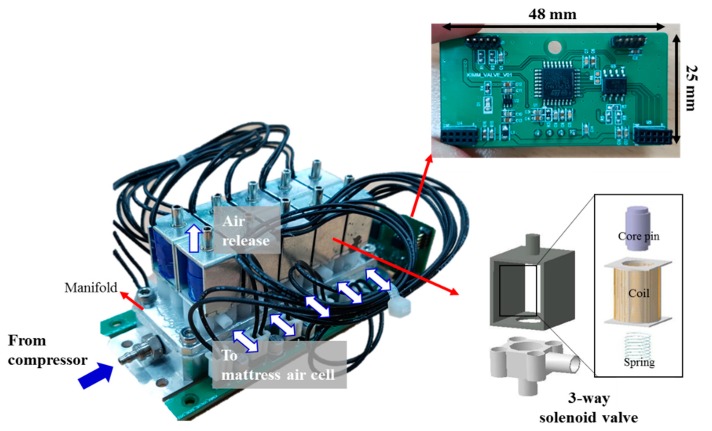
Pictures of the 3-way solenoid valves and a control board.

Figure 5 shows pictures of solenoid valves and their control board. The solenoid valve is an electromechanical device in which the solenoid uses an electric current to generate a magnetic field and thereby operate by a mechanism which regulates the opening of fluid flow in a valve [27]. In our system, each of the air-filled cells is connected to the respective solenoid valve. All solenoid valves were placed together on a common manifold as shown in Figure 5. The valve has the 3-way ports of dose, release, and distribution for air flow. If the valve is open, then an inlet port from compressor is connected to a distribution port to mattress air-filled cell, and air is injected to distribution port. If the valve is closed, then ports are isolated, and air is released through the exhaust port. The valve control board determines whether the electric current is passed through the solenoid, communicating with the main control unit. The solenoid valve consumes a current of 200 mA from a 5 V supply.

**Figure 6:**
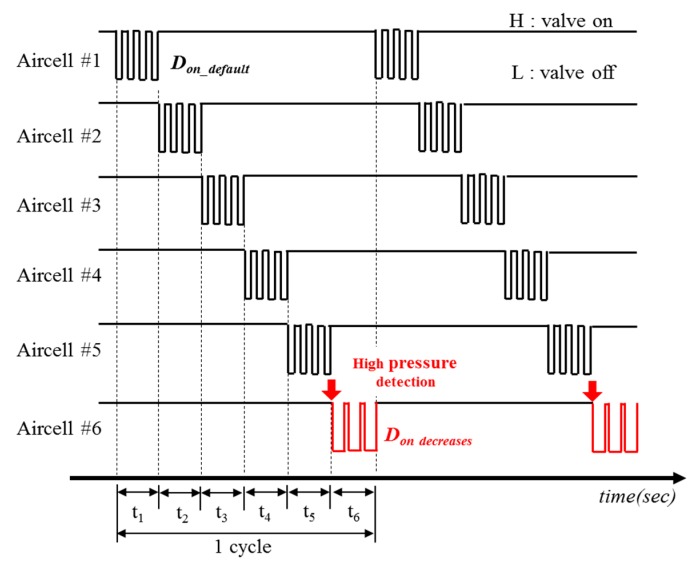
The timing diagram for solenoid valve control.

Figure 6 shows the timing diagram for a solenoid valve control. In the prototype, the 6 air-filled cells were alternately controlled during a cycle period. When the valve switch is ON with logic high, the valve is opened, and then air is injected from the compressor to air-filled cell, thereby inflating the air-filled cell. When the switch is OFF with logic low, the valve is closed, and the air is released from air-filled cell. The system sequentially determines the duration of inflation (or deflation) at the entrance of t_1_ – t_6_. For example, if the pressure level is at its lowest, *D_on_* of switches in solenoid valves is 50%, which means that the ON time for inflation is same with the OFF time for deflation, as shown in the air-filled cell #1-5 of Figure 6. If the higher pressure is detected, then the *D_on_* begins to decrease while at the same time increasing the deflation rate, as shown in the air-filled cell #6 of Figure 6. Therefore, the system is able to adaptively supply the corresponding air as the interface pressure changes, which results in the reducing effect in the interface pressure.

## 3. Results and Discussion

### 3.1. Performance of the TOF Optical Pressure Sensor

**Figure 7:**
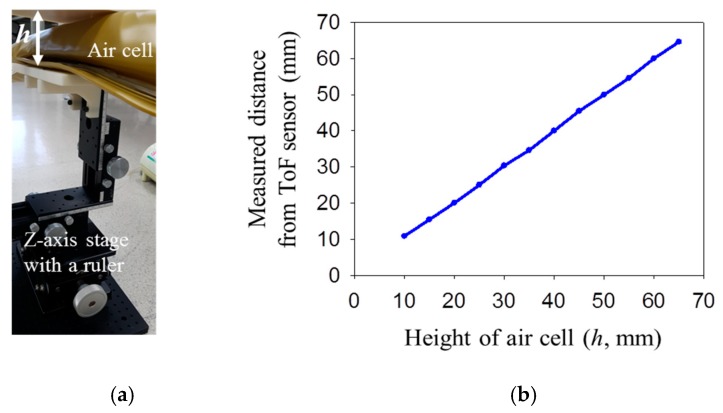
Measuring performance of the ToF optical sensor; (**a**) Setup for pressing the air-filled cell; (**b**) comparison of the measured distance by ToF optical sensor with actual height of air-filled cell by *Z*-axis stage.

The ToF optical pressure sensor is designed by positioning the commercial ToF optical sensor inside of air-filled cell. The ToF optical sensor can measure distance between bottom and top surface of air-filled cell. In Figure 7, the signals of the ToF optical sensor were compared with the height (*h*) of the air-filled cell after deformation. Figure 7a shows the *Z*-axis stage equipment that can precisely change the height of the air-filled cell in the vertical direction. Using the *Z*-axis stage equipment with a ruler, the air-filled cell was manually pressed while recording the actual height of air cell. The height of air cell was compared with the results from the ToF sensor in Figure 7b. The air-filled cell had a maximum height of 68 mm without deformation. The air cell was gradually pressed to reach the height of 10 mm. It is verified that the measured distance by the ToF optical sensor had an accuracy of ±0.57% compared with the actual height of the air-filled cell.

**Figure 8:**
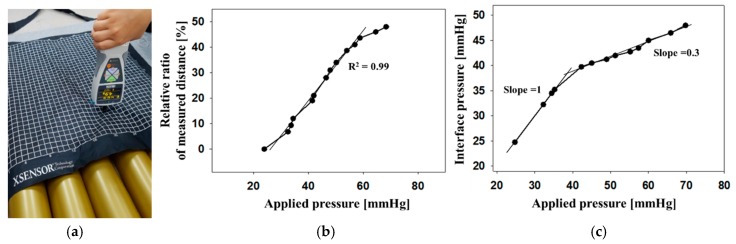
(**a**) Picture for measurement method, (**b**) the relative ratio in the measured distance and (**c**) the interface pressure at pressures applied by manual forces.

Figure 8 shows the calibration curve of the relative ratio in the measured distance at different pressures. The relative ratio in the measured distance is the ratio of the changed distance after deformation compared with initial distance. This relative value is effective to objectively evaluate the multiple sensors’ performance, excluding the common noise. Figure 8a shows the picture for the measurement method to validate the relation between the measured distance and the interface pressure. The commercial equipment X3Pro (XSENSOR, Calgary, AB, Canada) is placed on air-filled cells to investigate the interface pressure. And different forces were manually applied in downward direction using a push-pull gauge (IMADA, Japan). Figure 8b shows the relative ratio of measured distance as different pressures applied by a push-pull gauge. At the same time, the interface pressures were measured by X3Pro as shown in Figure 8c. Our system is capable of measuring the minimum pressure level of 24 mmHg, which is comparable to a commercialized sensor [11]. This system has a linear response under the interface pressure of 35 mmHg. The slope of the interface pressure to the applied pressure is calculated to be 0.3 over 35 mmHg.

### 3.2. Prototype of Active Body Pressure Relief System with the Optical Pressure Sensor

**Figure 9:**
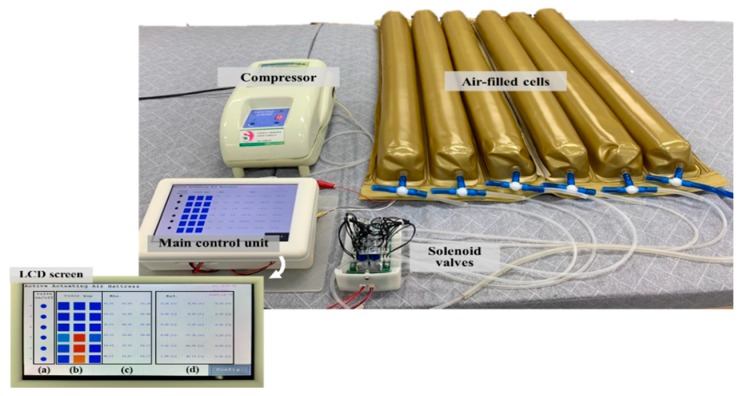
Picture of the prototype system including air-filled cells, solenoid valves, a main control unit and a compressor.

Figure 9 shows the prototype system including air-filled cells, solenoid valves, a main control unit, and a compressor. As a proof of concept, 6 air-filled cells were tested under the hip placement. The air-filled cells have the ToF optical sensors inside them. The main control unit receives the pressure signal from the ToF optical pressure sensor and regulates air flow by controlling the switches of solenoid valves. In particular, the main control unit has a 7-inch LCD to use for user’s interface while visualizing the body pressure distribution in real time. The inset in Figure 9 shows the LCD screen window. The screen shows sections of (a) valve ON and OFF status, (b) color map responding to the pressure level and the measured (c) absolute and (d) relative distance values. 

The optical pressure sensor could quantitatively measure the body pressure distribution when a participant was taking different postures on the mattress. Postures are as follows: (a) a supine position, (b) a left lateral position, (c) a right lateral position and (d) a sitting position. Table 1 shows the color visualization and the measured corresponding data at different postures, respectively. The pressure was defined as red color when the relative distance ratio was larger than 45%. The distance ratio of 0% was expressed with the blue color. The distance ratio was classified with a total of 1024 colors between the red and the blue color. In a supine position of posture at Table 1a, the sensors of index of 6 to 11 mainly responded to applied pressure. The sensor of index 9 was matched with the coccyx of the body bones representing the highest pressure with red color. As expected, in the left lateral position of posture at Table 1b, the left-side sensors of index 12 to 17 showed the largest changes in color. We could recognize the left pelvic bone from the colors in the sensors of index 15 and 16. In case of the right lateral position of posture at Table 1c, a participant was lying across the middle and right regions. Therefore, lower and even pressures were visualized due to the pressure redistribution. In (d) of Table 1, a participant was sitting on the mattress. Through the color changes in the sensors of index 3, 9 to 11 and 15, the coccyx and pelvic bones of body could be clearly recognized. In these experiments, the proposed system successfully quantified the body pressure distribution in real time. 

**Figure 10:**
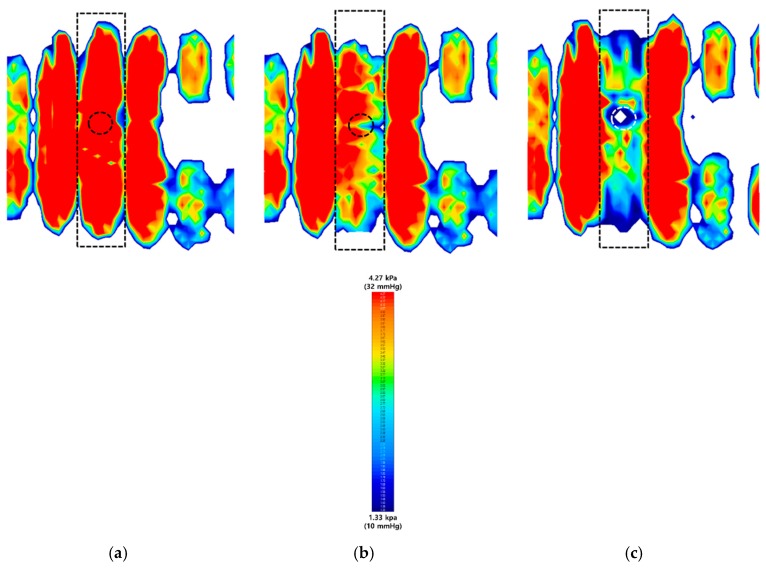
Description of changes in interface pressure, (**a**) without any air-flow control, (**b**) with a passive air-flow control, and (**c**) with an active air-flow control. These colors were visualized by commercial equipment of X3Pro.

**Figure 11:**
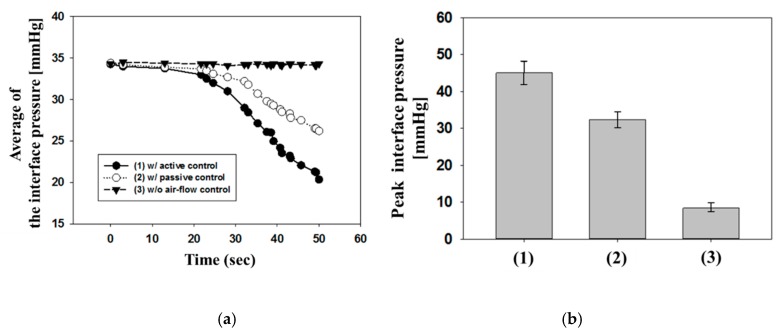
(**a**) The changes of the average pressure in a length of air cell over time and (**b**) peak pressure values at ToF sensor position at different air-flow controls of (1) without any air-flow control, (2) with a passive air-flow control, and (3) with an active air-flow control.

The pressure relieving effect was confirmed by investigating the interface pressure with commercial equipment X3Pro when a participant was taking a supine posture on the air-filled cells. In Figure 10, the changes in interface pressure without any flow control, with a passive air-flow control, and an active control mechanism were investigated, respectively, when the third one of air cells (square dashed line) is inflated and deflated. The pressure distribution was captured in 50 seconds. The red color represents the pressure level over than 32 mmHg. This value is the critical pressure level as a criterion for the occurrence of a pressure ulcer [28,29]. Figure 11a shows the changes of the average pressure in a length of air cell over time at different air-flow controls. In Figure 11b, the peak pressure values at ToF sensor position (circle dashed line) were compared (1) without any flow control, (2) with a passive air-flow control, and (3) an active flow control after 50 seconds. Without any air-flow control in Figure 10a, the surface area in the dashed line had an average pressure of 34.3 mmHg and peak pressure of 45 mmHg, which means that the pressure level may lead to the presence of pressure ulcer. In a passive air-flow control of Figure 10b, which equals to the *D_on_* of 50%, the interface pressure decreased to the average of 26.2 mmHg and peak pressure of 32.4 mmHg after 50 seconds. When air flow was actively regulated in response to the pressure level in Figure 10c, the interface pressure was reduced to an average of 20.3 mmHg and peak pressure of 8.4 mmHg. Here, a white spot in the center of Figure 10c means the pressure level of zero. Also, it is showing a rapid decrease of pressure compared with passive air-flow control. Consequently, the ability of the active controlled mattress was to achieve a more even and lower distribution of stress regions across the body. Therefore, the system successfully performed the active pressure relieving mechanism, maintaining the interface pressure at a low enough level to prevent the pressure ulcer.

## 4. Conclusions

In this study, an active body pressure relief system was developed to prevent pressure ulcer. The system is an alternating pressure air mattress with adaptive control of air flow in response to the pressure level. The system continuously quantifies the body pressure distribution by indirectly measuring the interface pressure with the ToF optical pressure sensor. This optical sensor is placed on the bottom surface of the air-filled cell. Non-contact characteristic of optical sensor contributes to improved durability and repeatability. Our system can effectively reduce the pressure stress through active control mechanism based on the negative feedback loop. If the interface pressure is higher, the body supporting air-filled cell is more deflated, then the pressure decreases again. It was demonstrated that the system successfully quantified the body pressure distribution at different postures. We could recognize the bones and bump regions of the body, although the prototype measured pressures in discrete regions. It was verified that the pressure relief mechanism performed well keeping the interface pressure low enough to prevent the pressure ulcer. In further work, we will arrange an array of air cells with high resolution expecting a fully relief of high pressure. The system can be potentially used for various bedding applications for measuring the body pressure distribution and relieving the pressure stress.

## Figures and Tables

**Table 1:** The color visualization and measured corresponding data when a participant was taking different postures on the mattress; (**a**) a supine position, (**b**) a left lateral position, (**c**) a right lateral position and (**d**) a sitting position.

Posture		Color Map	Measured Data
(a)	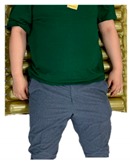	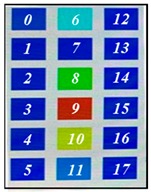	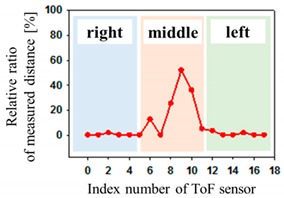
(b)	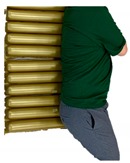	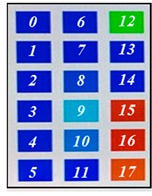	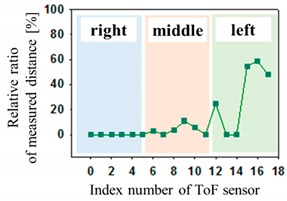
(c)	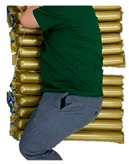	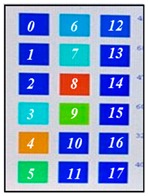	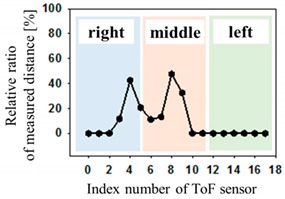
(d)